# Variability of the Transferrin Receptor 2 Gene in AMD

**DOI:** 10.1155/2014/507356

**Published:** 2014-02-06

**Authors:** Daniel Wysokinski, Janusz Blasiak, Mariola Dorecka, Marta Kowalska, Jacek Robaszkiewicz, Elzbieta Pawlowska, Jerzy Szaflik, Jacek Pawel Szaflik

**Affiliations:** ^1^Department of Molecular Genetics, University of Lodz, Pomorska 141/143, 90-236 Lodz, Poland; ^2^Department of Ophthalmology, Medical University of Silesia, Ceglana 35, 40-514 Katowice, Poland; ^3^Laser Centrum Okulistyczne, ul. Boya 4A/24, 00-621 Warsaw, Poland; ^4^Department of Ophthalmology, Medical University of Warsaw, ul. Lindleya 4, 02-005 Warsaw, Poland; ^5^Department of Developmental Dentistry, Medical University of Lodz, Pomorska 251, 92-216 Lodz, Poland; ^6^Department of Ophthalmology, Medical University of Warsaw and Samodzielny Publiczny Kliniczny Szpital Okulistyczny, Sierakowskiego 13, 03-709 Warsaw, Poland

## Abstract

Oxidative stress is a major factor in the pathogenesis of age-related macular degeneration (AMD). Iron may catalyze the Fenton reaction resulting in overproduction of reactive oxygen species. Transferrin receptor 2 plays a critical role in iron homeostasis and variability in its gene may influence oxidative stress and AMD occurrence. To verify this hypothesis we assessed the association between polymorphisms of the *TFR2* gene and AMD. A total of 493 AMD patients and 171 matched controls were genotyped for the two polymorphisms of the *TFR2* gene: c.1892C>T (rs2075674) and c.−258+123T>C (rs4434553). We also assessed the modulation of some AMD risk factors by these polymorphisms. The CC and TT genotypes of the c.1892C>T were associated with AMD occurrence but the latter only in obese patients. The other polymorphism was not associated with AMD occurrence, but the CC genotype was correlated with an increasing AMD frequency in subjects with BMI < 26. The TT genotype and the T allele of this polymorphism decreased AMD occurrence in subjects above 72 years, whereas the TC genotype and the C allele increased occurrence of AMD in this group. The c.1892C>T and c.−258+123T>C polymorphisms of the *TRF2* gene may be associated with AMD occurrence, either directly or by modulation of risk factors.

## 1. Introduction

Age-related macular degeneration is the leading cause of irreversible vision loss among elderly in the developed world and accounts for about half of all newly registered blindness cases. At the advanced stage AMD develops to one of two clinically distinct forms—dry or wet [1]. The dry (atrophic) form of AMD occurs in about 90% of patients and its outcome is an accumulation of drusen in the central region of the macula, between the retinal pigment epithelium (RPE) layer and the Bruch's membrane. The wet (neovascular or exudative AMD) form of AMD occurs only in 10% of patients; however, it is the most common cause of irreversible vision loss in this disease. This form is characterized by choroidal neovascularization leading to leakages and bleeding into the retina [2]. Local inflammatory process develops and central disciform scar is formed. Photoreceptors and retinal pigment epithelium degenerate, leading to the loss of central vision [2].

Oxidative stress plays a major role in AMD pathophysiology [3]. The retina is exposed to oxidative stress from endogenous and exogenous sources, which may exceed its oxidative defense capacity, causing retinal cell death [4].

The presence of free iron ions may enhance the level of oxidative stress through the Fenton reaction, which requires hydrogen peroxide, produced in the cell in several physiological processes [5]. *Post mortem* studies showed a higher concentration of iron in RPE layer and Bruch's membrane in AMD subjects than in non-AMD individuals [6]. Similarly, in a case study of patient suffering from aceruloplasminemia, a disease resulting from a defect in the ceruloplasmin gene, an accumulation of iron in the macula was found and features of AMD developed [7].

Cells absorb most of iron through the cooperation of transferrin (Tf) and its receptors 1 and 2 (TfR and TfR2) [8]. An elevated expression of both *TF* and *TFR2 *genes was found to be associated with AMD [9]. *TFR2* is also involved in nontransferrin bound-iron uptake and plays an important role in iron homeostasis regulation [10, 11]. Moreover, a number of mutations in *TFR2* were reported to influence iron homeostasis leading to iron overload disease—hemochromatosis type III [12]. The association of AMD occurrence and family history of this disease confirms the importance of the hereditary component of AMD [13]. It is thought that genotype-environment interaction plays an important role in AMD incidence [14]. Therefore, we hypothesize that variations in *TRF2*, an important gene of iron metabolism, may influence the extent of oxidative stress and play a role in AMD pathogenesis. In the present work we checked for the association between two polymorphisms of the *TRF2* gene: c.1892C>T (rs2075674) and c.–258+123T>C (rs4434553) and AMD as well as modulation of this association by some AMD risk factors.

## 2. Materials and Methods

### 2.1. Subjects

Six hundred two individuals were enrolled in this study: 493 AMD patients (311 in wet form of the disease and 182 in its dry form) and 171 controls. The control group comprised of age- and sex-matched individuals routinely examined with clinically excluded AMD and other retinal diseases. No one patient reported any genetic disease. Every patient was subjected to an ophthalmic examination, including best-corrected visual acuity, intraocular pressure, slit lamp examination, and fundus examination, performed with a slit lamp equipped with either noncontact or contact fundus lenses. Diagnosis of AMD was confirmed by optical coherence tomography (OCT) and, in some cases, by fluorescein angiography (FA) and indocyanine green angiography (ICG). OCT evaluated retinal thickness, the presence of RPE atrophy, drusen, or subretinal fluid and intraretinal edema; angiography assessed the anatomical status of the retinal vessels and the presence of choroidal neovascularization and leakage. The criteria for patients' selection were based on the clinical usefulness; dry form corresponded to AREDS category 2, 3, and 4 (geographic atrophy subtype) while wet form to AREDS category 4 (choroidal neovascularization or neovascularmaculopathy subtype) [15]. The OCT examinations were performed with Stratus OCT model 3000, software version 4.0 (Oberkochen). The FA and ICG examinations were completed with a Topcon TRC-50I IX fundus camera equipped with the digital Image Net image system, version 2.14 (Topcon). Structured questionnaire was used in order to obtain information from all patients regarding their lifestyle habits and family/personal history of AMD. Venous blood samples were obtained following the written consent from the patients and the Bioethics Committee of the Medical University of Warsaw, Poland.

### 2.2. DNA Isolation

Genomic DNA was obtained from peripheral blood samples with the use of AxyPrep Blood Genomic DNA Miniprep kit (Axygen Biosciences) with all the steps performed according to the manufacturer's protocol. Samples of DNA were frozen at −20°C until use.

### 2.3. Genotype Determination

**Figure 1:**
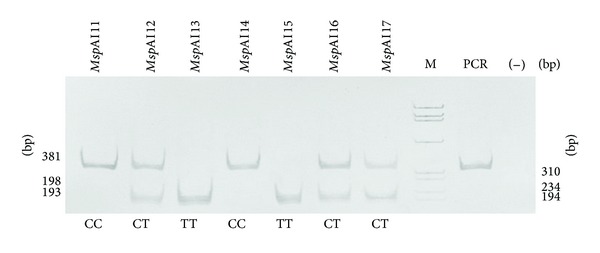
Representative picture of transferrin receptor polymorphism (c.1892C>T) analysis. The first lane (M) is DNA mass marker *φ*X-174 DNA BsuRI. Digested PCR products were separated on a 10% polyacrylamide gel and stained with ethidium bromide.

**Figure 2:**
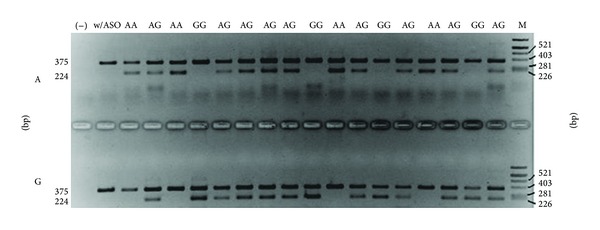
Representative picture of transferrin receptor polymorphism (c.–258+123T>C) analysis using allele-specific oligonucleotides PCR application. The first lane is negative control (no template), w/ASO is flanking (constitutive) primers only, and last lane (M) is DNA mass marker *φ*X-174 DNA BsuRI. The upper row is the A allele-specific primer and lower row is the G allele-specific primer.

Genotyping of the c.1892C>T polymorphism was performed with restriction fragment length polymorphism polymerase chain reaction (RFLP-PCR). The reaction tube contained genomic DNA, 250 nM of each primer, 50 nM dNTP mix, 1.5 mM MgCl_2_, and 1.25 U Taq polymerase (Biotools) in PCR reaction buffer (100 mM Tris-HCl, pH 8.3, 500 mM KCl, and 0.1% gelatin). The sequences of primers are shown in Table 2. Thermal cycling conditions were initial denaturation at 95°C for 5 min, 29 cycles: denaturation at 95°C for 30 s, annealing at 56°C for 30 s, and elongation at 72°C for 1 min. Amplified fragments were digested for 4 h with the restriction enzyme* Msp*A1I (Fermentas) (Table 1) and the samples were separated on an 8% polyacrylamide gel (Figure 1). For the genotype determination of the polymorphic site (c.−258+123T>C, rs4434553) in *TFR2* the allele-specific oligonucleotide polymerase chain reaction (ASO-PCR) was used. Reaction tubes contained genomic DNA, polymerase buffer, 0.25 *μ*M of each primer (forward flanking, reverse flanking primer, and ASO-primer) (Sigma-Aldrich), 0.5 *μ*M dNTPs, 1.5 *μ*M MgCl_2_, 1 U/*μ*L polymerase (BIOTOOLS B&M Labs), and H_2_O. Sequences of primers and lengths of DNA fragments are shown in Table 3. PCR was run on BIO-RAD C1000 Thermal Cycler (BIO-RAD Laboratories). Thermal cycling conditions were initial denaturation at 95°C for 3 min, 33 cycles: denaturation at 95°C for 30 s, annealing at 62°C for 30 s, elongation at 72°C for 1 min, and final extension at 72°C for 3 min. The amplified DNA fragments were separated in 1.8% agarose gel (HR agarose, BIOTOOLS B&M Labs), Tris-borate-EDTA buffer at 5 V/cm. The pBR322 DNA/AluI digest was used as a mass marker (Figure 2). The gels were stained with ethidium bromide and analyzed by the digital imaging system InGenius Bio Imaging (Syngene).

### 2.4. Serum Iron Assay

Venous blood samples were collected and centrifuged. Serum samples were portioned and stored frozen (*‒*20°C). Before use samples were centrifuged at 12 000 ×g for 15 min. Serum iron level has been determined by QuantiChrom Iron Assay kit (Bioassay Systems) according to the manufacturer's protocol. The absorbance was read at 450 nm.

### 2.5. Data Analysis

Hardy-Weinberg equilibrium (HWE) was checked using the *χ* ^2^ test for each group. The allelic frequencies were calculated by gene counting and genotypes were scored. The significance of the differences between distributions of genotypes and alleles was tested using the *χ* ^2^ analysis. Multiple logistic regression analysis was run to assess the association between the genotypes and alleles of the polymorphisms and AMD incidence. The genotype-associated risk was expressed by crude odds ratio with 95% confidence intervals and the *P* value. Odds ratios were then adjusted for possible interfering factors. To verify a potential gene-environment interaction, the patients and controls were stratified depending on age (<72 years and ≥72 years), sex, living environment (rural/urban), smoking status (never/former/current and never/moderate/heavy), and BMI (<26/26–30/>30). Multiple logistic regression analyses were run to test the association of genotypes and environmental and social factors with AMD occurrence. To establish polymorphism influence on AMD form progression (wet/dry), the OR analysis was performed between both groups. The serum iron level was compared between AMD and control group using Mann-Whitney rank sum test. To assess the potential association between genotypes of polymorphisms and the iron level the 1-way ANOVA analysis was performed. Statistical analysis was performed using Statistica 9.0 package (Statsof).

## 3. Results

**Figure 3:**
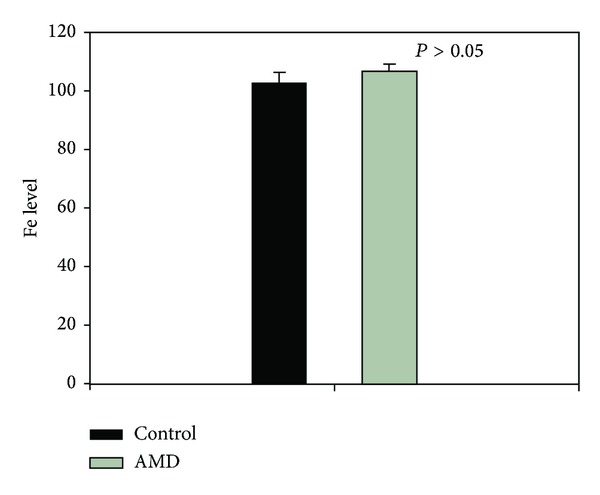
Mean concentration of serum iron level [*μ*g/dL] in control and AMD group assessed colorimetrically. Control group is black bar and AMD patients is gray bar. One hundred and seventy one persons were analyzed for the control, and 662 were analyzed for AMD.

Initially we performed a genotype-independent analysis between those two groups of the environmental and social factors of confirmed or possible role in AMD. Those included age, BMI, smoking, family history of AMD (among 1st degree relatives), and environment of living (rural/urban) (Table 2). We found no association between AMD occurrence and BMI, smoking status, or the environment of living. However, an association was found for age and family AMD history. Next we analyzed the frequencies of the genotypes and alleles of polymorphisms in the *TFR2* gene: c.1892C>T and c.–258+123T>C in controls and AMD patients as well as in the group with the dry and wet forms of AMD. We found that the CC genotype of the c.1892C>T polymorphism was negatively correlated with the incidence of AMD (Table 3). We did not find any correlation between the c.–258+123T>C polymorphism and AMD occurrence either for general AMD or for dry/wet form (data not shown). We then compared the frequencies of genotypes and alleles in the dry versus wet AMD groups to detect influence of the polymorphisms on AMD progression expressed as its transition from dry to wet form. We found that none of the genotypes/alleles of either polymorphism altered the risk of the progression of AMD (data not shown). In order to analyze the gene-environment interaction, we stratified the groups depending on the potential risk factors. We found that the TT genotype of the c.1892C>T polymorphism had a protective effect against AMD occurrence for patients with BMI > 30 (Table 4), whereas the CC genotype of the c.–258+123T>C polymorphism was associated with a decrease in AMD occurrence for individuals with BMI > 26 (Table 5). Moreover, the odds ratio analysis has shown that the TT genotype and the T allele reduced AMD occurrence in patients younger than 72 years, and the CT genotype and the C allele increased the risk. None of the genotypes/alleles altered AMD occurrence in patients aged 71 years and more (Table 6). Next, we compared the serum iron concentrations between AMD patients and controls. We did not observe any difference between these groups (Figure 3). We did not detect any association between the genotypes of both polymorphisms and iron concentration in serum.

## 4. Discussion

An exceptionally high metabolism rate in the retina together with the highest oxygen consumption and oxygen tension makes this organ especially prone to oxidative stress [16]. Moreover, the retina is constantly exposed to visible light, including high-energy blue light, which may generate ROS in photochemical reactions [17]. Photoreceptor outer segments are rich in polyunsaturated fatty acids (PUFA), which may be involved in the autoxidation cascade [18]. Similarly, retinal lipofuscin may undergo peroxidation and damage adjacent tissues [19]. Also catabolic processes in the retina generates ROS. Lipofuscin, and other photosensitizers, including melanin and rhodopsin, may absorb high-energy photons leading to photochemical reactions with generation of radical species [18]. Several lines of evidence suggest that an excess of iron, resulting from perturbations in iron homeostasis, may contribute to oxidative stress, which may play a role in numerous pathological conditions [20–22], including AMD [23]. Since transferrin-transferrin receptors iron uptake system seems to be of a special importance for iron balance in the organism, and numerous data indicate that deregulation of that system may be deleterious, it seems possible that it may also play a role in AMD pathogenesis. Our previous data suggest that transferrin gene variation may contribute to AMD, and transferrin level may differ between AMD and controls [24]. Therefore, it may be expected that the gene encoding transferrin receptor 2 may also be associated with this condition. Disturbances in the *TFR2* gene expression may result in cellular damage directly related to oxidative stress [25, 26].

We chose two polymorphisms in *TFR2*, which may have a phenotypic manifestation. The c.1892C>T polymorphism is a synonymous change p.Ala617. Using open-access SNP annotation tools we assessed that this change may be involved in splicing regulation—a T variant may create the site for exonic splicing repressor (hnRNP-H) binding [27]. The other polymorphism, positioned 5′-upstream, c.–258+123T>C, is located inside the binding site for the GATA-1 transcription factor [28], which is known to regulate *TFR2* expression [29]. We found that in our group, despite age being an *ex definitione *key risk factor for AMD, also positive family history was independently associated with AMD occurrence, and this is consistent with numerous previous reports, suggesting a strong genetic basis of AMD. Unexpectedly, we found no correlation between AMD and smoking status, although we speculate that this association may fall beyond the sensitivity threshold of our experiment, mainly because of not enough number of smoking-positive responses in the questionnaire. Next, we performed an OR analysis to search for a direct association between genotypes and alleles of the polymorphisms and AMD occurrence. We found no association between c.–258+123T>C and AMD occurrence. For the c.1892C>T polymorphism we found that the common variant CC may be associated with a lower frequency of AMD occurrence, although this association was significant in crude OR only, and becomes nonsignificant after adjusting for age and sex. Therefore, this association remains uncertain and requires further analysis. Moreover, the minor, T/T, variant of the c.1892C>T polymorphism may have protective role among individuals with BMI above 30. We observed also a protective effect of the CC genotype of the c.–258+123T>C polymorphism in the subgroup of low BMI value (<26). Overweight and obesity are often reported to play a role in AMD pathogenesis [30]. We did not obtain results indicating the BMI as an independent risk factor for AMD, but we detected a gene-BMI interplay as a potential risk predictor for AMD and such interactions have been reported elsewhere [31]. Another association was observed between the c.–258+123T>C polymorphism and age—the group aged below 72 years; the common TT genotype of this polymorphism might have a protective role against AMD occurrence, while the rare CC variant might enhance the occurrence of the disease. These results may suggest that at a young age the influence of genetic factors may be more prominent in AMD pathogenesis, whereas later the age-related processes may play a central role. Lastly, we found no significant difference between serum iron levels in AMD and control group, so we concluded that the process of AMD pathogenesis and possible disturbances in iron homeostasis may not cause changes in serum iron level. However it is also possible that such change may be specific for the eye. This aspect seems especially interesting and further searching for iron level differences between particular organs/systems would bring significant progress in defining the role of iron in AMD.

## 5. Conclusions

In this study we presented the data suggesting that variants of the *TFR2* gene may play a role in the modulation of AMD risk. We reported the interaction effect between polymorphisms of the *TFR2* gene with BMI and age. These results suggest an important role of iron homeostasis in AMD pathogenesis.

## Figures and Tables

**Table 1:** Sequences of primers and restriction enzymes for genotyping of the c.−258+123T>C and c.1892C>T polymorphisms of the *TRF2 *gene.

Polymorphism	Sequences of primers			
c.−258+123T>C (rs4434553)	Flanking	F: 5′ GATCACATGGGTTTCTACCTCTTT R: 5′ CTTAGGTCTCAGGGGAGCAG		PCR^a^ 375 bp
	ASO	R-A spec: 5′ GACCTGAGTAGGAGCTGATGTTC R-G spec: 5′ GACCTGAGTAGGAGCTGATGTTT		ASO^b^ 224 bp

	Sequences of primers	Enzyme (target allele)	Target sequence	Genotypes^c^

c.1892C>T (rs2075674)	F: 5′-ACTCCCTGCCGTCGAGTTCT-3′ R: 5′-ACTCCCAGAGACCAAGAGCG-3′	*MspA*1I (T)	5′…C(A/C)G↓C(G/T)G…3′ 3′…G(T/G)C↑G(C/A)C…3′	CC 381 CT 381/198/193 TT 198/193

F: forward primer. R: reverse primer. ^a^PCR product length using only flanking primers. ^b^PCR product length with ASO reverse primers. ^c^Restriction products lengths (bp) for each genotype.

**Table 2:** The association of AMD with age, BMI, tobacco smoking, family history of AMD, and living environment.

Factor	AMD	
	OR^1)^ (95% CI)	*P* ^2)^
Age^3)^	**1.02 (1.00–1.04)↑**	**<0.05**
BMI	0.99 (0.94–1.03)	—
Smoking (ever)	0.88 (0.6–1.28)	—
Family AMD	**8.88 (2.73**–**28.84)↑**	**<0.001**
Living enviromment^5^	0.69 (0.4–1.19)	—

^1)^Odds ratio with 95% confidence interval. ^2)^ *χ* ^2^ test. ^3)^For +1 year. ^4)^Adjusted to females. ^5)^Adjusted to rural. Data in boldface are statistically significant.

**Table 3:** Distribution of genotypes, frequency of alleles of the c.1892C>T polymorphism of the *TFR2 *gene, and odds ratios (OR) with 95% confidence intervals (95% CI) in age-related macular degeneration (AMD) patients and controls.

Genotype/allele c.1892C>T	Control (*N* = 171)	AMD (*N* = 493)	OR^1)^ (95% CI)	*P* ^2)^	OR^3)^ (95% CI)	*P* ^2)^
	*N* (%)	*N* (%)				
**CC**	97 (0.57)	267 (0.54)	**0.42 (0.32–0.54)↓**	**<0.001**	0.86 (0.6–1.23)	0.411
CT	54 (0.32)	183 (0.37)	1.28 (0.88–1.85)	0.196	1.35 (0.93–1.97)	0.118
TT	20 (0.12)	43 (0.09)	0.72 (0.41–1.27)	0.288	0.71 (0.4–1.26)	0.246
C	248 (0.73)	717 (0.73)	1.01 (0.77–1.33)	0.920	0.99 (0.76–1.29)	0.920
T	94 (0.27)	269 (0.27)	0.99 (0.75–1.3)	0.920	1.01 (0.78–1.33)	0.920

^1)^Crude odds ratio with 95% confidence interval; ^2)^ *χ* ^2^ test;^ 3)^odds ratio adjusted for age and sex.

Data in boldface are statistically significant.

**Table 4:** Distribution of genotypes, frequency of alleles of the c.1892C>T polymorphism of the *TFR2 *gene, and odds ratios (OR) with 95% confidence intervals (95% CI) in age-related macular degeneration (AMD) patients and controls with respect to body mass index (BMI) (<26; 26–30; >30).

Genotype/allele c.1892C>T	BMI <26			
	Control (63) Number (%)	AMD (153) Number (%)	OR^1)^ (95% CI)	*P* ^2)^
CC	38 (0.6)	78 (0.51)	0.66 (0.36–1.21)	0.180
CT	20 (0.32)	55 (0.36)	1.19 (0.63–2.24)	0.588
TT	5 (0.08)	20 (0.13)	1.94 (0.69–5.49)	0.210
C	96 (0.76)	211 (0.69)	0.7 (0.45–1.1)	0.121
T	30 (0.24)	95 (0.31)	1.43 (0.91–2.25)	0.121

Genotype/allele c.1892C>T	BMI 26–30			
	Control (43) Number (%)	AMD (146) Number (%)	OR^1)^ (95% CI)	*P* ^2)^

CC	25 (0.58)	78 (0.53)	0.74 (0.37–1.51)	0.413
CT	13 (0.30)	55 (0.38)	1.66 (0.78–3.54)	0.192
TT	5 (0.12)	13 (0.09)	0.64 (0.2–1.98)	0.432
C	63 (0.73)	211 (0.72)	0.93 (0.54–1.59)	0.778
T	23 (0.27)	81 (0.28)	1.08 (0.63–1.86)	0.778

Genotype/allele c.1892C>T	BMI >30			
	Control (39) Number (%)	AMD (77) Number (%)	OR^1)^ (95% CI)	*P* ^2)^

CC	22 (0.56)	42 (0.55)	0.85 (0.38–1.87)	0.683
CT	10 (0.26)	32 (0.42)	2.32 (0.96–5.58)	0.061
**TT**	7 (0.18)	3 (0.04)	**0.19 (0.04**–**0.78)↓**	**0.022**
C	54 (0.69)	116 (0.75)	1.26 (0.7–2.29)	0.441
T	24 (0.31)	38 (0.25)	0.79 (0.44–1.44)	0.441

^1)^Adjusted for age and sex; ^2)^ *χ* ^2^; data in boldface are statistically significant.

**Table 5:** Distribution of genotypes, frequency of alleles of the c.−258+123T>C polymorphism of the *TFR2 *gene, and odds ratios (OR) with 95% confidence intervals (95% CI) in age-related macular degeneration (AMD) and controls with respect to body mass index (BMI) (<26; 26–30; >30).

Genotype/allele c. −258+123T>C	BMI <26			
	Control (63) Number (%)	AMD (153) Number (%)	OR^1)^ (95% CI)	*P* ^2)^
TT	22 (0.35)	55 (0.36)	1.05 (0.57–1.96)	0.870
TC	24 (0.38)	74 (0.48)	1.57 (0.85–2.88)	0.149
**CC**	17 (027)	24 (0.16)	**0.48 (0.23**–**0.98)↓**	**0.044**
T	68 (0.54)	184 (0.60)	1.29 (0.86–1.95)	0.224
C	58 (0.46)	122 (0.40)	0.77 (0.51–1.17)	0.224

Genotype/allele c. −258+123T>C	BMI 26–30			
	Control (43) Number (%)	AMD (144) Number (%)	OR^1)^ (95% CI)	*P* ^2)^

TT	14 (0.33)	50 (0.35)	1.11 (0.53–2.33)	0.790
TC	19 (0.44)	63 (0.44)	1.03 (0.51–2.09)	0.937
CC	10 (0.23)	31 (0.22)	0.84 (0.36–1.9)	0.685
T	47 (0.55)	163 (0.57)	1.1 (0.69–1.77)	0.690
C	39 (0.45)	125 (0.43)	0.91 (0.57–1.46)	0.690

Genotype/allele c. −258+123T>C	BMI >30			
	Control (171) Number (%)	AMD (77) Number (%)	OR^1)^ (95% CI)	*P* ^2)^

TT	60 (0.35)	21 (0.27)	0.69 (0.3–1.58)	0.373
TC	75 (0.43)	39 (0.51)	1.16 (0.53–2.55)	0.710
CC	38 (0.22)	17 (0.22)	1.32 (0.49–3.53)	0.580
T	193 (0.56)	81 (0.53)	0.78 (0.45–1.35)	0.374
C	149 (0.44)	73 (0.47)	1.28 (0.74–2.23)	0.374

^1)^Adjusted for age and sex; ^2)^ *χ* ^2^ test; data in boldface are statistically significant.

**Table 6:** Distribution of genotypes, frequency of alleles of the c.258+123T>C polymorphism of the *TFR2* gene, and odds ratios (OR) with 95% confidence intervals (95% CI) in age-related macular degeneration (AMD) and controls in two age groups (<72 and ≥72).

Genotype/allele c.−258+123T>C	Age							
	<72				≥72			
	Control (*N* = 87)	AMD (*N* = 227)	OR^1)^ (95% CI)	*P* ^2)^	Control (*N* = 83)	AMD (*N* = 247)	OR^1)^ (95% CI)	*P* ^2)^
	*N* (%)	*N* (%)			*N* (%)	*N* (%)		
**TT**	38 (0.44)	76 (0.33)	**0.33 (0.15**–**0.73)↓**	**0.006**	21 (0.25)	80 (0.32)	1.79 (0.73–4.39)	0.203
**TC**	33 (0.38)	101 (0.44)	**2.57 (1.11**–**5.96)↑**	**0.028**	40 (0.48)	123 (0.50)	0.94 (0.43–2.09)	0.887
CC	16 (0.18)	50 (0.22)	1.41 (0.55–3.64)	—	22 (0.27)	44 (0.18)	0.54 (0.21–1.41)	0.209
**T**	109 (0.63)	253 (0.56)	**0.56 (0.33**–**0.95)↓**	**0.031**	82 (0.49)	283 (0.57)	1.57 (0.89–2.78)	0.123
**C**	65 (0.37)	201 (0.44)	**1.79 (1.05**–**3.03)↑**	**0.031**	84 (0.51)	211 (0.43)	0.64 (0.36–1.13)	0.123

^1)^Odds ratio adjusted for age and living environment; ^2)^ *χ* ^2^ test; data in boldface are statistically significant.

## Abbreviations

AMD: Age-related macular degeneration
RPE: Retinal pigment epithelium
OCT: Optical coherence tomography
FA: Fluorescein angiography
ICG: Indocyanine green angiography
AREDS: Age-Related Eye Disease Study
RFLP-PCR: Restriction fragment length polymorphism polymerase chain reaction
ASO-PCR: Allele-specific oligonucleotide polymerase chain reaction
HWE: Hardy-Weinberg equilibrium
OR: Odds ratio
BMI: Body mass index
UV: Ultraviolet
ROS: Reactive oxygen species
PUFA: Polyunsaturated fatty acids
SD: Standard deviation.
